# Pre-pregnancy Maternal Weight and Gestational Weight Gain Increase the Risk for Childhood Asthma and Wheeze: An Updated Meta-Analysis

**DOI:** 10.3389/fped.2020.00134

**Published:** 2020-04-03

**Authors:** Shufang Liu, Bo Zhou, Yunfeng Wang, Kundi Wang, Zhixin Zhang, Wenquan Niu

**Affiliations:** ^1^Graduate School, Beijing University of Chinese Medicine, Beijing, China; ^2^Department of Pediatrics, China-Japan Friendship Hospital, Beijing, China; ^3^International Medical Services, China-Japan Friendship Hospital, Beijing, China; ^4^Institute of Clinical Medical Sciences, China-Japan Friendship Hospital, Beijing, China

**Keywords:** asthma, gestational weight gain, meta-analysis, maternal obesity, maternal overweight

## Abstract

**Background:** Mounting evidence suggests that childhood asthma is closely associated with maternal weight before pregnancy and gestational weight gain (GWG), yet the results are not often reproducible.

**Objectives:** We conducted a comprehensive meta-analysis, aiming to evaluate the association of pre-pregnancy maternal obesity or overweight and high GWG with the risk for childhood asthma and wheeze.

**Methods:** Literature search, quality assessment, and data extraction were completed independently and in duplicate. Effect-size estimates are expressed as odds ratio (OR) with 95% confidence interval (CI).

**Results:** Twenty-two observational studies involving 145,574 mother-child pairs were meta-analyzed. In overall analyses, maternal obesity or overweight in pre-pregnancy significantly increased the risk of both childhood asthma and wheeze (adjusted OR: 1.41 and 1.13, 95% CI: 1.26–1.59 and 1.07–1.20, both *p* < 0.001). Per 1 kg/m^2^ increment in maternal body mass index was associated with a significantly increased risk of childhood asthma and wheeze (adjusted OR: 1.03, 95% CI: 1.02-1.03, *p* < 0.001). Compared with normal GWG, very high GWG (adjusted OR: 1.24, 95% CI: 1.04-1.47, *p*: 0.018), moderate high GWG (adjusted OR: 1.12, 95% CI: 1.04-1.21, *p*: 0.004), and very low GWG (adjusted OR: 1.26, 95% CI: 1.08-1.47, *p*: 0.004) increased the risk of childhood asthma and wheeze. There was a low probability of publication bias.

**Conclusions:** Our findings indicate that both pre-pregnancy maternal obesity or overweight and very to moderate high or low GWG render their offspring susceptible to a significantly increased risk of having childhood asthma and wheeze.

## Introduction

Childhood asthma is the most common chronic respiratory disease, and it has reached epidemic proportions worldwide (1). Global statistics show that death rates from asthma in children range from 0 to 0.7 per 100,000 people (1). As reported by Loftus and Wise, 8.4% of persons in the United States have asthma as compared with 4.3% of the population worldwide, and both numbers are on the rise (2). It is worth noting that the average annual asthma prevalence is higher in children (9.5%) than adults (7.7%) (2). Thus, new tools to early identify children who are at risk for asthma development and could be targeted for preventive strategies are imperative to improve global health.

Evidence is mounting suggesting that childhood asthma is closely associated with maternal weight before pregnancy and gestational weight gain (GWG) (3, 4). Pre-pregnancy maternal obesity is increasingly recognized as a major public health issue worldwide. For instance, epidemiological data from the United States recorded that the prevalence of pre-pregnancy maternal obesity was 13% in 1993 and 22% in 2003, which reflects a roughly 69% increase (5). Numerous studies have examined the association between intrauterine exposure to maternal obesity or gestational weight gain and the risk of childhood asthma, with inconsistent and inconclusive findings (6–8). Forno and colleagues in 2014 interrogated summary data from 14 studies, and found that pre-pregnancy maternal obesity was associated with the significant risk of both ever and current asthma or wheeze in children, and significance was only noticed for the association between high GWG and ever asthma or wheeze (9). Given the accumulating data afterwards (4, 7, 10–15), there is a need to reevaluate this association in a more comprehensive manner.

To yield more information, we conducted an updated meta-analysis to test the hypothesis that pre-pregnancy maternal obesity or overweight and high GWG are associated with an increased risk of asthma and wheeze in children, and meanwhile we attempted to explore the possible causes of between-study heterogeneity via subgroup and meta-regression analyses.

## Methods

This meta-analysis of the available literature was conducted according to the Preferred Reporting Items for Systematic reviews and Meta-Analyses (PRISMA) statement (16). The PRISMA checklist is presented in Supplementary Table 1.

### Search Strategy

The PubMed, Excerpt Medica Database (EMBASE), Cochrane Central Register of Controlled Trials (CENTRAL), Cochrane Database of Systematic Reviews, and Google Scholar were searched from inception to July 30, 2019 for observational studies that assessed the association between pre-pregnancy maternal obesity or overweight and GWG and the risk for asthma or wheeze in children.

The following medical subject headings (MESH) were used for literature search, and they are expressed in the Boolean form: (maternal OR mother OR parental OR pre-pregnancy OR pregnancy OR gestational) AND (obesity OR obese OR overweight OR body mass index OR BMI OR percent body fat OR PBF OR body weight OR anthropometry OR fat OR fatness OR adiposity OR weight gain OR body fat OR body fat composition) AND (child OR children OR childhood OR infant OR adolescent OR young OR youth OR teenage) AND (asthma OR wheeze OR asthma like symptoms OR atopic disease OR atopy OR airway hyperresponsiveness OR respiratory symptom). The bibliographies of identified articles were also searched for additional references.

### Eligible Criteria

Studies were included if they fulfilled the following criteria: (i) study design: observational studies of either nested or cross-sectional case-control design; (ii) study participants: children with outcomes measured from birth to under 18 years of age, and mothers with body mass index (BMI) reported at the beginning of pregnancy or at certain point during pregnancy or with GWG reported at the end of pregnancy; (iii) study outcomes: asthma and/or wheeze; (iv) diagnoses: parental report or doctor diagnosis or medical records; (v) language: articles published in the English language. In case of study outcomes recorded at multiple time points, only outcome at the latest time point was abstracted. In a nested case-control study, cases of a disease that occur in a defined cohort are identified as the “case” group, and, for each case, a specified number of matched controls are selected from among those in the cohort who have not developed the disease by the time of disease occurrence in the case as the “control” group (17).

Articles were excluded if they were published in form of conference abstract, case report, case series, letter to the editor or correspondence, editorial, or review.

### Data Extraction

The eligibility of each retrieved article was independently evaluated by two authors (S.L. and B.Z.) according to the afore mentioned inclusion and exclusion criteria, and disagreement was adjudicated by a third author (Z.Z.). The same two authors extracted data from qualified articles independently, and typed them into separate databases, including surname of the first author, year of publication, country where the study was conducted, study type, sample size, study outcomes, diagnoses of asthma and/or wheeze, effect size estimates (both adjusted and unadjusted), and baseline characteristics of study children if available, with any discrepancies resolved through discussion. The inter-rater agreement was high as revealed by the kappa statistic, which equaled to 0.99.

## Quality Assessment

The Newcastle-Ottawa Scale (NOS) (18) was adopted for quality assessment of each qualified study. In brief, the NOS contains eight items, which are categorized into three dimensions, including selection, comparability, and outcome or exposure. The NOS ranges from zero to nine stars, with more stars indicating higher quality. Study quality was independently assessed by two authors (S.L. and B.Z.), and areas of disagreement or uncertainty were resolved by consensus.

## Statistical Analyses

Only outcomes of interest provided by two or more studies are pooled and presented. Categorical BMI included obesity (BMI >30 kg/m^2^), overweight (BMI: 25-30 kg/m^2^), and underweight (BMI <18.5 kg/m^2^). Categorical GWG included very low GWG (GWG <5 kg), low GWG (GWG: 5-9 kg), high GWG (GWG: 15-20 kg); moderate high GWG (GWG: 20-25 kg), and very high GWG (GWG >25 kg). Effect size estimates are presented as odds ratio (OR) with 95% confidence interval (95% CI).

Statistical heterogeneity was judged using the inconsistency index (*I*^2^), and significant heterogeneity was reported if the *I*^2^ is over 50% (19). In the absence of heterogeneity, fixed-effects model is adopted, and in the presence of heterogeneity, random-effects model is adopted. In case of significant heterogeneity, both fixed-effects and random-effects models yield similar results, and so random-effects model is employed irrespective of the magnitude of heterogeneity. In addition, random-effects model was used owing to assumption of clinical and methodological diversity among the studies, which often leads to statistical heterogeneity. Possible causes of between-study heterogeneity were explored by means of subgroup and meta-regression analyses.

Publication bias was visually judged by both Begg's and filled funnel plots (20). The symmetry of funnel plots was appraised by the Egger's test. The trim-and-fill method was used to estimate the number of potentially missing studies stemming from publication bias (21). Significant publication bias was recorded if the probability of the Egger's test is <10%.

Statistical analyses were completed using the STATA software Release 14.1 for Windows (Stata Corp, College Station, TX).

## Results

### Qualified Studies

**Figure 1:**
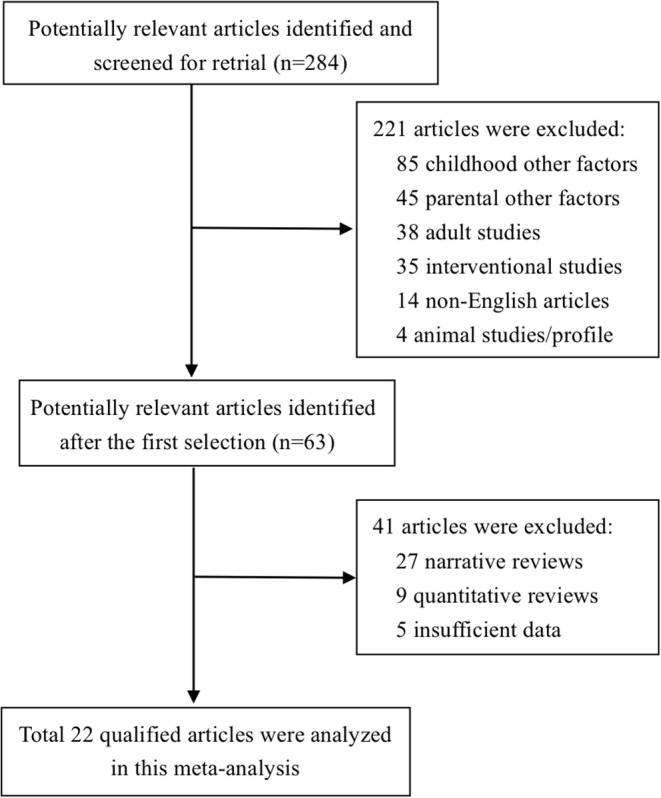
Flow diagram of search strategy and study exclusion with specific reasons.

Using predefined MESH terms, a total of 284 articles were identified and evaluated for eligibility. Based on the titles or abstracts, 221 articles were excluded due to obvious reasons. After reviewing the full texts of remaining 63 articles, 41 articles were further excluded, leaving 22 articles that fulfilled predetermined eligibility criteria in this meta-analysis (4, 7, 8, 10–15, 22–34). A flow diagram illustrating the exclusion of articles with specific reasons is shown in Figure 1.

### Baseline Characteristics of Qualified Studies

The baseline characteristics of 22 observational studies (2 cross-sectional and 20 nested case-control studies) involving 145,574 mother-child pairs were showed in Table 1. The average age of children ranged from 4 months to 16 years. Sixteen studies reported maternal categorical BMI before or at the beginning of pregnancy (4, 7, 8, 11–15, 24–28, 30, 32, 34), and thereof ten studies additionally reported maternal continuous BMI in pre-pregnancy (7, 10, 11, 14, 15, 27–29, 32, 33). Seven studies reported GWG, with one study reporting continuous GWG (32), and six reporting categorical GWG (4, 7, 8, 22, 23, 31). Three studies reported categorical GWG according to Institute of Medicine criteria (7, 14, 33).

**Table 1:** The baseline characteristics of individual studies involved in this meta-analysis.

**References**	**Maternal weight**	**Continent**	**Study type**	**Sample size**	**Male**	**Age**	**Weight modality**	**Diagnosis of asthma**	**Clinical endpoint**	**Comparison**	**ES**	**cES**	**aES**
Oliveti et al. (26)	GWG <9 kg	America	Cross-sectional	262	0.634	6.7	Self-report	Doctor-diagnosis	Asthma	Low GWG vs. normal GWG	OR	4.4	3.42
Rusconi et al. (23)	GWG <9 kg	Europe	Cross-sectional	15,609	0.512	6.7	Self-report	Self-report	Persistent wheeze	Low GWG vs. normal GWG	OR	1.09	1.08
Rusconi et al. (23)	GWG >15 kg	Europe	Cross-sectional	15,609	0.512	6.7	Self-report	Self-report	Persistent wheeze	High GWG vs. normal GWG	OR	1.23	1.2
Reichman et al. (24)	BMI ≥30 kg/m2	America	Longitudinal	1,971	0.524	3	Medical records	Doctor-diagnosis	Asthma	High BMI vs. normal BMI	OR	1.51	1.34
Haberg et al. (25)	BMI: 25–30 kg/m2	Europe	Longitudinal	33,192	NA	1.5	Self-report	Self-report	Wheeze	High BMI vs. normal BMI	OR	0.96	1.02
Haberg et al. (25)	BMI ≥30 kg/m2	Europe	Longitudinal	33,192	NA	1.5	Self-report	Self-report	Wheeze	High BMI vs. normal BMI	OR	1.21	1.15
Kumar et al. (26)	BMI: 25–30 kg/m2	America	Longitudinal	1,191	0.483	3	Self-report	Self-report	Recurrent wheeze	High BMI vs. normal BMI	OR	1.48	1.58
Kumar et al. (26)	BMI ≥30 kg/m2	America	Longitudinal	1,191	0.483	3	Self-report	Self-report	Recurrent wheeze	High BMI vs. normal BMI	OR	3.24	3.51
Scholtens et al. (27)	Continuous BMI	Europe	Longitudinal	3,963	NA	8	Self-report	Self-report	Asthma	Per BMI increment	OR	NA	0.98
Scholtens et al. (27)	Continuous BMI	Europe	Longitudinal	3,963	NA	8	Self-report	Self-report	Wheeze	Per BMI increment	OR	NA	1.01
Scholtens et al. (27)	Continuous BMI	Europe	Longitudinal	3,963	NA	8	Self-report	Self-report	Asthma	Per BMI increment	OR	NA	1.05
Scholtens et al. (27)	Continuous BMI	Europe	Longitudinal	3,963	NA	8	Self-report	Self-report	Wheeze	Per BMI increment	OR	NA	1.06
Scholtens et al. (27)	BMI >25 kg/m2	Europe	Longitudinal	3,963	NA	8	Self-report	Self-report	Asthma	High BMI vs. normal BMI	OR	1	0.86
Scholtens et al. (27)	BMI >25 kg/m2	Europe	Longitudinal	3,963	NA	8	Self-report	Self-report	Wheeze	High BMI vs. normal BMI	OR	1.1	1.06
Scholtens et al. (27)	BMI >25 kg/m2	Europe	Longitudinal	3,963	NA	8	Self-report	Self-report	Asthma	High BMI vs. normal BMI	OR	1.7	1.52
Scholtens et al. (27)	BMI >25 kg/m2	Europe	Longitudinal	3,963	NA	8	Self-report	Self-report	Wheeze	High BMI vs. normal BMI	OR	1.58	1.44
Patel et al. (28)	BMI: 25–30 kg/m2	Europe	Longitudinal	6,945	0.515	15.5	Medical records	Doctor-diagnosis	Ever wheeze	High BMI vs. normal BMI	OR	1.31	1.18
Patel et al. (28)	BMI: 25–30 kg/m2	Europe	Longitudinal	6,945	0.515	15.5	Medical records	Doctor-diagnosis	Current wheeze	High BMI vs. normal BMI	OR	1.22	1.13
Patel et al. (28)	BMI <19 kg/m2	Europe	Longitudinal	6,945	0.515	15.5	Medical records	Doctor-diagnosis	Ever wheeze	Low BMI vs. normal BMI	OR	0.91	0.8
Patel et al. (28)	BMI <19 kg/m2	Europe	Longitudinal	6,945	0.515	15.5	Medical records	Doctor-diagnosis	Current wheeze	Low BMI vs. normal BMI	OR	1.07	0.87
Patel et al. (28)	BMI ≥30 kg/m2	Europe	Longitudinal	6,945	0.515	15.5	Medical records	Doctor-diagnosis	Ever wheeze	High BMI vs. normal BMI	OR	1.07	0.99
Patel et al. (28)	BMI ≥30 kg/m2	Europe	Longitudinal	6,945	0.515	15.5	Medical records	Doctor-diagnosis	Current wheeze	High BMI vs. normal BMI	OR	1.52	1.54
Patel et al. (28)	Continuous BMI	Europe	Longitudinal	6,945	0.515	15.5	Medical records	Doctor-diagnosis	Ever wheeze	Per BMI increment	OR	1.03	1.03
Patel et al. (28)	Continuous BMI	Europe	Longitudinal	6,945	0.515	15.5	Medical records	Doctor-diagnosis	Current wheeze	Per BMI increment	OR	1.04	1.05
Caudri et al. (29)	Continuous BMI	Europe	Longitudinal	3,963	0.516	8	Self-report	Self-report	Persistent wheeze	Per BMI increment	OR	NA	1.06
Guerra et al. (30)	BMI: 25–30 kg/m2	Europe	Longitudinal	1,107	0.511	1.17	Self-report	Self-report	Persistent wheeze	High BMI vs. normal BMI	RR	1.3	1
Guerra et al. (30)	BMI <19 kg/m2	Europe	Longitudinal	1,107	0.511	1.17	Self-report	Self-report	Persistent wheeze	Low BMI vs. normal BMI	RR	1.5	1.7
Guerra et al. (30)	BMI ≥30 kg/m2	Europe	Longitudinal	1,107	0.511	1.17	Self-report	Self-report	Persistent wheeze	High BMI vs. normal BMI	RR	4.1	4.2
Halonen et al. (31)	GWG >18.6 kg	America	Longitudinal	261	NA	9	Medical records	Doctor-diagnosis	Asthma	High GWG vs. normal GWG	OR	3.4	3.4
Halonen et al. (31)	BMI <18.5 kg/m2	Europe	Longitudinal	38,874	0.51	7	Self-report	Doctor-diagnosis	Asthma	Low BMI vs. normal BMI	OR	1.11	1.03
Halonen et al. (31)	BMI <18.5 kg/m2	Europe	Longitudinal	38,874	0.51	7	Self-report	Doctor-diagnosis	Ever asthma	Low BMI vs. normal BMI	OR	NA	1.02
Halonen et al. (31)	BMI <18.5 kg/m2	Europe	Longitudinal	38,874	0.51	7	Self-report	Doctor-diagnosis	Current asthma	Low BMI vs. normal BMI	OR	NA	1.07
Halonen et al. (31)	BMI <18.5 kg/m2	Europe	Longitudinal	38,874	0.51	7	Self-report	Doctor-diagnosis	Persistent wheeze	Low BMI vs. normal BMI	OR	0.91	0.81
Halonen et al. (31)	BMI: 25–30 kg/m2	Europe	Longitudinal	38,874	0.51	7	Self-report	Doctor-diagnosis	Ever asthma	High BMI vs. normal BMI	OR	NA	1.19
Halonen et al. (31)	BMI: 25–30 kg/m2	Europe	Longitudinal	38,874	0.51	7	Self-report	Doctor-diagnosis	Current asthma	High BMI vs. normal BMI	OR	NA	1.24
Halonen et al. (31)	BMI: 30–35 kg/m2	Europe	Longitudinal	38,874	0.51	7	Self-report	Doctor-diagnosis	Persistent wheeze	High BMI vs. normal BMI	OR	1.78	1.62
Halonen et al. (31)	BMI: 25–30 kg/m2	Europe	Longitudinal	38,874	0.51	7	Self-report	Doctor-diagnosis	Persistent wheeze	High BMI vs. normal BMI	OR	1.28	1.19
Halonen et al. (31)	BMI: 25–30 kg/m2	Europe	Longitudinal	38,874	0.51	7	Self-report	Doctor-diagnosis	Asthma	High BMI vs. normal BMI	OR	1.24	1.22
Halonen et al. (31)	BMI: 30–35 kg/m2	Europe	Longitudinal	38,874	0.51	7	Self-report	Doctor-diagnosis	Asthma	High BMI vs. normal BMI	OR	1.6	1.56
Halonen et al. (31)	BMI: 30–35 kg/m2	Europe	Longitudinal	38,874	0.51	7	Self-report	Doctor-diagnosis	Ever asthma	High BMI vs. normal BMI	OR	NA	1.5
Halonen et al. (31)	BMI: 30–35 kg/m2	Europe	Longitudinal	38,874	0.51	7	Self-report	Doctor-diagnosis	Current asthma	High BMI vs. normal BMI	OR	NA	1.58
Halonen et al. (31)	BMI ≥35 kg/m2	Europe	Longitudinal	38,874	0.51	7	Self-report	Doctor-diagnosis	Asthma	High BMI vs. normal BMI	OR	1.64	1.55
Halonen et al. (31)	BMI ≥35 kg/m2	Europe	Longitudinal	38,874	0.51	7	Self-report	Doctor-diagnosis	Ever asthma	High BMI vs. normal BMI	OR	NA	1.55
Halonen et al. (31)	BMI ≥35 kg/m2	Europe	Longitudinal	38,874	0.51	7	Self-report	Doctor-diagnosis	Current asthma	High BMI vs. normal BMI	OR	NA	1.48
Halonen et al. (31)	BMI ≥35 kg/m2	Europe	Longitudinal	38,874	0.51	7	Self-report	Doctor-diagnosis	Persistent wheeze	High BMI vs. normal BMI	OR	1.6	1.44
Halonen et al. (31)	GWG: 16–19 kg	Europe	Longitudinal	38,874	0.51	7	Self-report	Doctor-diagnosis	Asthma	High GWG vs. normal GWG	OR	0.96	0.97
Halonen et al. (31)	GWG: 16–19 kg	Europe	Longitudinal	38,874	0.51	7	Self-report	Doctor-diagnosis	Ever asthma	High GWG vs. normal GWG	OR	NA	1.03
Halonen et al. (31)	GWG: 16–19 kg	Europe	Longitudinal	38,874	0.51	7	Self-report	Doctor-diagnosis	Current asthma	High GWG vs. normal GWG	OR	NA	0.9
Halonen et al. (31)	GWG: 16–19 kg	Europe	Longitudinal	38,874	0.51	7	Self-report	Doctor-diagnosis	Persistent wheeze	High GWG vs. normal GWG	OR	0.96	0.99
Halonen et al. (31)	GWG: 20–24 kg	Europe	Longitudinal	38,874	0.51	7	Self-report	Doctor-diagnosis	Asthma	Moderate high GWG vs. normal GWG	OR	1.18	1.15
Halonen et al. (31)	GWG: 20–24 kg	Europe	Longitudinal	38,874	0.51	7	Self-report	Doctor-diagnosis	Ever asthma	Moderate high GWG vs. normal GWG	OR	NA	1.13
Halonen et al. (31)	GWG: 20–24 kg	Europe	Longitudinal	38,874	0.51	7	Self-report	Doctor-diagnosis	Current asthma	Moderate high GWG vs. normal GWG	OR	NA	1.15
Halonen et al. (31)	GWG: 20–24 kg	Europe	Longitudinal	38,874	0.51	7	Self-report	Doctor-diagnosis	Persistent wheeze	Moderate high GWG vs. normal GWG	OR	1	0.96
Halonen et al. (31)	GWG: 5–9 kg	Europe	Longitudinal	38,874	0.51	7	Self-report	Doctor-diagnosis	Asthma	Low GWG vs. normal GWG	OR	1.13	1.02
Halonen et al. (31)	GWG: 5–9 kg	Europe	Longitudinal	38,874	0.51	7	Self-report	Doctor-diagnosis	Ever asthma	Low GWG vs. normal GWG	OR	NA	0.94
Halonen et al. (31)	GWG: 5–9 kg	Europe	Longitudinal	38,874	0.51	7	Self-report	Doctor-diagnosis	Current asthma	Low GWG vs. normal GWG	OR	NA	1.11
Halonen et al. (31)	GWG: 5–9 kg	Europe	Longitudinal	38,874	0.51	7	Self-report	Doctor-diagnosis	Persistent wheeze	Low GWG vs. normal GWG	OR	1.41	1.24
Halonen et al. (31)	GWG <5 kg	Europe	Longitudinal	38,874	0.51	7	Self-report	Doctor-diagnosis	Asthma	Very low GWG vs. normal GWG	OR	1.62	1.17
Halonen et al. (31)	GWG <5 kg	Europe	Longitudinal	38,874	0.51	7	Self-report	Doctor-diagnosis	Ever asthma	Very low GWG vs. normal GWG	OR	NA	1.54
Halonen et al. (31)	GWG <5 kg	Europe	Longitudinal	38,874	0.51	7	Self-report	Doctor-diagnosis	Current asthma	Very low GWG vs. normal GWG	OR	NA	0.84
Halonen et al. (31)	GWG <5 kg	Europe	Longitudinal	38,874	0.51	7	Self-report	Doctor-diagnosis	Persistent wheeze	Very low GWG vs. normal GWG	OR	1.2	0.82
Halonen et al. (31)	GWG ≥25 kg	Europe	Longitudinal	38,874	0.51	7	Self-report	Doctor-diagnosis	Asthma	Very high GWG vs. normal GWG	OR	1.27	1.19
Halonen et al. (31)	GWG ≥25 kg	Europe	Longitudinal	38,874	0.51	7	Self-report	Doctor-diagnosis	Ever asthma	Very high GWG vs. normal GWG	OR	NA	1.11
Halonen et al. (31)	GWG ≥25 kg	Europe	Longitudinal	38,874	0.51	7	Self-report	Doctor-diagnosis	Current asthma	Very high GWG vs. normal GWG	OR	NA	1.23
Halonen et al. (31)	GWG ≥25 kg	Europe	Longitudinal	38,874	0.51	7	Self-report	Doctor-diagnosis	Persistent wheeze	Very high GWG vs. normal GWG	OR	1.19	1.12
Leermakers et al. (32)	BMI: 25–30 kg/m2	Europe	Longitudinal	4,656	0.5	4	Self-report	Self-report	Wheeze	High BMI vs. normal BMI	OR	NA	1.04
Leermakers et al. (32)	BMI: 25–30 kg/m2	Europe	Longitudinal	4,656	0.5	4	Self-report	Self-report	Wheeze	High BMI vs. normal BMI	OR	NA	1.09
Leermakers et al. (32)	BMI: 25–30 kg/m2	Europe	Longitudinal	4,656	0.5	4	Self-report	Self-report	Wheeze	High BMI vs. normal BMI	OR	NA	0.95
Leermakers et al. (32)	BMI <20 kg/m2	Europe	Longitudinal	4,656	0.5	4	Self-report	Self-report	Wheeze	Low BMI vs. normal BMI	OR	NA	1.02
Leermakers et al. (32)	BMI <20 kg/m2	Europe	Longitudinal	4,656	0.5	4	Self-report	Self-report	Wheeze	Low BMI vs. normal BMI	OR	NA	0.93
Leermakers et al. (32)	BMI <20 kg/m2	Europe	Longitudinal	4,656	0.5	4	Self-report	Self-report	Wheeze	Low BMI vs. normal BMI	OR	NA	1.19
Leermakers et al. (32)	BMI ≥30 kg/m2	Europe	Longitudinal	4,656	0.5	4	Self-report	Self-report	Wheeze	High BMI vs. normal BMI	OR	NA	1.1
Leermakers et al. (32)	BMI ≥30 kg/m2	Europe	Longitudinal	4,656	0.5	4	Self-report	Self-report	Wheeze	High BMI vs. normal BMI	OR	NA	0.93
Leermakers et al. (32)	BMI ≥30 kg/m2	Europe	Longitudinal	4,656	0.5	4	Self-report	Self-report	Wheeze	High BMI vs. normal BMI	OR	NA	1.41
Leermakers et al. (32)	Continuous BMI	Europe	Longitudinal	4,656	0.5	4	Self-report	Self-report	Wheeze	Per BMI increment	OR	NA	1.01
Leermakers et al. (32)	Continuous BMI	Europe	Longitudinal	4,656	0.5	4	Self-report	Self-report	Wheeze	Per BMI increment	OR	NA	1.01
Pike et al. (33)	Continuous BMI	Europe	Longitudinal	940	0.516	6	Medical records	Both	Ever wheeze	Per BMI increment	RR	1.02	1.01
Pike et al. (33)	Continuous BMI	Europe	Longitudinal	940	0.516	6	Medical records	Both	Ever asthma	Per BMI increment	RR	1.01	1
Pike et al. (33)	Continuous BMI	Europe	Longitudinal	940	0.516	6	Medical records	Both	Current wheeze	Per BMI increment	RR	1	1
Pike et al. (33)	Continuous BMI	Europe	Longitudinal	940	0.516	6	Medical records	Both	Current asthma	Per BMI increment	RR	1.02	1.02
Pike et al. (33)	Continuous BMI	Europe	Longitudinal	940	0.516	6	Medical records	Both	Persistent wheeze	Per BMI increment	RR	1.02	1.02
Pike et al. (33)	Excessive	Europe	Longitudinal	940	0.516	6	Medical records or Measure	Both	Ever wheeze	High GWG vs. normal GWG	RR	NA	0.96
Pike et al. (33)	Excessive	Europe	Longitudinal	940	0.516	6	Medical records or Measure	Both	Ever asthma	High GWG vs. normal GWG	RR	NA	0.99
Pike et al. (33)	Excessive	Europe	Longitudinal	940	0.516	6	Medical records or Measure	Both	Current wheeze	High GWG vs. normal GWG	RR	NA	0.83
Pike et al. (33)	Excessive	Europe	Longitudinal	940	0.516	6	Medical records or Measure	Both	Current asthma	High GWG vs. normal GWG	RR	NA	1.05
Pike et al. (33)	Excessive	Europe	Longitudinal	940	0.516	6	Medical records or Measure	Both	Persistent wheeze	High GWG vs. normal GWG	RR	NA	0.94
Pike et al. (33)	Inadequate	Europe	Longitudinal	940	0.516	6	Medical records or Measure	Both	Ever wheeze	Low GWG vs. normal GWG	RR	NA	0.98
Pike et al. (33)	Inadequate	Europe	Longitudinal	940	0.516	6	Medical records or Measure	Both	Ever asthma	Low GWG vs. normal GWG	RR	NA	1.15
Pike et al. (33)	Inadequate	Europe	Longitudinal	940	0.516	6	Medical records or Measure	Both	Current wheeze	Low GWG vs. normal GWG	RR	NA	0.80
Pike et al. (33)	Inadequate	Europe	Longitudinal	940	0.516	6	Medical records or Measure	Both	Current asthma	Low GWG vs. normal GWG	RR	NA	1.24
Pike et al. (33)	Inadequate	Europe	Longitudinal	940	0.516	6	Medical records or Measure	Both	Persistent wheeze	Low GWG vs. normal GWG	RR	NA	0.91
Wright et al. (33)	BMI ≥30 kg/m2	America	Longitudinal	261	0.49	2	Medical records	Self-report	Recurrent wheeze	High BMI vs. normal BMI	OR	2.61	2.56
de Vries et al. (10)	Continuous BMI	Europe	Longitudinal	4,860	0.499	0.28	Self-report	Self-report	Wheeze	Per BMI increment	OR	1.03	1.03
Ekstrom et al. (11)	BMI: 25–29.9 kg/m2	Europe	Longitudinal	3,294	0.505	16	Medical records	Self-report	Asthma	High BMI vs. normal BMI	OR	NA	1.14
Ekstrom et al. (11)	BMI <18.5 kg/m2	Europe	Longitudinal	3,294	0.505	16	Medical records	Self-report	Asthma	Low BMI vs. normal BMI	OR	NA	1.29
Ekstrom et al. (11)	BMI ≥30 kg/m2	Europe	Longitudinal	3,294	0.505	16	Medical records	Self-report	Asthma	High BMI vs. normal BMI	OR	NA	1.53
Ekstrom et al. (11)	Continuous BMI	Europe	Longitudinal	3,294	0.505	16	Medical records	Self-report	Asthma	Per BMI increment	OR	NA	1.06
Harskamp-van et al. (15)	BMI: 25–29.9 kg/m2	Europe	Longitudinal	3,185	NA	7.5	Self-report	Doctor-diagnosis	Current wheeze	High BMI vs. normal BMI	RR	0.93	0.9
Harskamp-van et al. (15)	BMI: 25–29.9 kg/m2	Europe	Longitudinal	3,185	NA	7.5	Self-report	Doctor-diagnosis	Ever asthma	High BMI vs. normal BMI	RR	1.33	1.38
Harskamp-van et al. (15)	BMI <18.5 kg/m2	Europe	Longitudinal	3,185	NA	7.5	Self-report	Doctor-diagnosis	Current wheeze	Low BMI vs. normal BMI	RR	1.15	1.32
Harskamp-van et al. (15)	BMI <18.5 kg/m2	Europe	Longitudinal	3,185	NA	7.5	Self-report	Doctor-diagnosis	Ever asthma	Low BMI vs. normal BMI	RR	1.64	1.42
Harskamp-van et al. (15)	BMI ≥30 kg/m2	Europe	Longitudinal	3,185	NA	7.5	Self-report	Doctor-diagnosis	Current wheeze	High BMI vs. normal BMI	RR	2.09	2.15
Harskamp-van et al. (15)	BMI ≥30 kg/m2	Europe	Longitudinal	3,185	NA	7.5	Self-report	Doctor-diagnosis	Ever asthma	High BMI vs. normal BMI	RR	2.72	2.24
Dumas et al. (4)	BMI: 25–29.9 kg/m2	America	Longitudinal	12,963	0.462	11.5	Self-report	Doctor-diagnosis	Asthma	High BMI vs. normal BMI	OR	1.27	1.19
Dumas et al. (4)	BMI <20.0 kg/m2	America	Longitudinal	12,963	0.462	11.5	Self-report	Doctor-diagnosis	Asthma	Low BMI vs. normal BMI	OR	1.08	1.05
Dumas et al. (4)	BMI ≥30 kg/m2	America	Longitudinal	12,963	0.462	11.5	Self-report	Doctor-diagnosis	Asthma	High BMI vs. normal BMI	OR	1.48	1.34
Dumas et al. (4)	GWG: 15.9–20 kg	America	Longitudinal	12,963	0.462	11.5	Self-report	Doctor-diagnosis	Asthma	High GWG vs. normal GWG	OR	1.09	1.04
Dumas et al. (4)	GWG: 6.8–10.9 kg	America	Longitudinal	12,963	0.462	11.5	Self-report	Doctor-diagnosis	Asthma	Low GWG vs. normal GWG	OR	1.08	1.07
Dumas et al. (4)	GWG <6.8 kg	America	Longitudinal	12,963	0.462	11.5	Self-report	Doctor-diagnosis	Asthma	Very low GWG vs. normal GWG	OR	1.51	1.28
Dumas et al. (4)	GWG ≥20.4 kg	America	Longitudinal	12,963	0.462	11.5	Self-report	Doctor-diagnosis	Asthma	Moderate high GWG vs. normal GWG	OR	1.16	1.05
Taylor-Robinson et al. (13)	Obesity	Europe	Longitudinal	11,141	0.509	7	Self-report	Self-report	Persistent wheeze	High BMI vs. normal BMI	RRR	1.38	1.27
Taylor-Robinson et al. (13)	Overweight	Europe	Longitudinal	11,141	0.509	7	Self-report	Self-report	Persistent wheeze	High BMI vs. normal BMI	RRR	1.25	1.22
Taylor-Robinson et al. (13)	Underweight	Europe	Longitudinal	11,141	0.509	7	Self-report	Self-report	Persistent wheeze	Low BMI vs. normal BMI	RRR	1.1	1.02
Polinski et al. (7)	BMI: 25–29.9 kg/m2	America	Longitudinal	6,450	0.489	4	Self-report	Doctor-diagnosis	Asthma	High BMI vs. normal BMI	OR	1.26	1.22
Polinski et al. (7)	BMI <18.5 kg/m2	America	Longitudinal	6,450	0.489	4	Self-report	Doctor-diagnosis	Asthma	Low BMI vs. normal BMI	OR	1.18	1.07
Polinski et al. (7)	BMI ≥30 kg/m2	America	Longitudinal	6,450	0.489	4	Self-report	Doctor-diagnosis	Asthma	High BMI vs. normal BMI	OR	1.8	1.5
Polinski et al. (7)	Continuous BMI	America	Longitudinal	6,450	0.489	4	Self-report	Doctor-diagnosis	Asthma	Per BMI increment	OR	1.03	1.03
Polinski et al. (7)	GWG: 16–19 kg	America	Longitudinal	6,450	0.489	4	Self-report	Doctor-diagnosis	Asthma	High GWG vs. normal GWG	OR	0.79	0.84
Polinski et al. (7)	GWG: 20–24 kg	America	Longitudinal	6,450	0.489	4	Self-report	Doctor-diagnosis	Asthma	Moderate high GWG vs. normal GWG	OR	1.14	1.16
Polinski et al. (7)	GWG: 5–9 kg	America	Longitudinal	6,450	0.489	4	Self-report	Doctor-diagnosis	Asthma	Low GWG vs. normal GWG	OR	1.25	1.06
Polinski et al. (7)	GWG <5 kg	America	Longitudinal	6,450	0.489	4	Self-report	Doctor-diagnosis	Asthma	Very low GWG vs. normal GWG	OR	2.2	1.56
Polinski et al. (7)	GWG ≥25 kg	America	Longitudinal	6,450	0.489	4	Self-report	Doctor-diagnosis	Asthma	Very high GWG vs. normal GWG	OR	1.71	1.53
Polinski et al. (7)	Excessive	America	Longitudinal	6,450	0.489	4	Self-report	Doctor-diagnosis	Asthma	High GWG vs. normal GWG	OR	0.95	0.85
Polinski et al. (7)	Inadequate	America	Longitudinal	6,450	0.489	4	Self-report	Doctor-diagnosis	Asthma	Low GWG vs. normal GWG	OR	1.13	0.99
Rajappan et al. (14)	BMI <18.5 kg/m2	Europe	Longitudinal	2,799	0.516	1	Medical records	Self-report	Wheeze	Low BMI vs. normal BMI	RR	0.7	0.71
Rajappan et al. (14)	BM ≥30 kg/m2	Europe	Longitudinal	2,799	0.516	1	Medical records	Self-report	Wheeze	High BMI vs. normal BMI	RR	1.19	1.19
Rajappan et al. (14)	BMI: 25–29.99 kg/m2	Europe	Longitudinal	2,799	0.516	1	Medical records	Self-report	Wheeze	High BMI vs. normal BMI	RR	1.08	1.11
Rajappan et al. (14)	Continuous BMI	Europe	Longitudinal	2,799	0.516	1	Medical records	Self-report	Wheeze	Per BMI increment	RR	1.02	1.02
Rajappan et al. (14)	Excessive	Europe	Longitudinal	2,799	0.516	1	Medical records or Measure	Self-report	Wheeze	High GWG vs. normal GWG	RR	NA	0.97
Rajappan et al. (14)	Inadequate	Europe	Longitudinal	2,799	0.516	1	Medical records or Measure	Self-report	Wheeze	Low GWG vs. normal GWG	RR	NA	1.05
Goudarzi et al. (15)	BMI: 25–29.9 kg/m2	Asia	Longitudinal	3,296	0.516	7	Self-report	Self-report	Wheeze	High BMI vs. normal BMI	RR	NA	1.11
Goudarzi et al. (15)	BMI <18.5 kg/m2	Asia	Longitudinal	3,296	0.516	7	Self-report	Self-report	Wheeze	Low BMI vs. normal BMI	RR	NA	0.69
Goudarzi et al. (15)	BMI ≥30 kg/m2	Asia	Longitudinal	3,296	0.516	7	Self-report	Self-report	Wheeze	High BMI vs. normal BMI	RR	NA	1.67
Goudarzi et al. (15)	Continuous BMI	Asia	Longitudinal	3,296	0.516	7	Self-report	Self-report	Wheeze	Per BMI increment	RR	1.03	1.03

*ES, effect size; cES, crude effect size; aES, adjusted effect size; GWG, gestational weight gain; BMI, body mass index; NA, not available*.

### Quality Assessment

Using the NOS system, the total stars of 2 cross-sectional case-control studies were 6 and 4, respectively (Supplementary Table 2). For nested case-control studies, the total stars ranged from 5 to 8 (mean: 6.45, standard deviation: 0.83) (Supplementary Table 3).

### Overall Analyses

**Figure 2:**
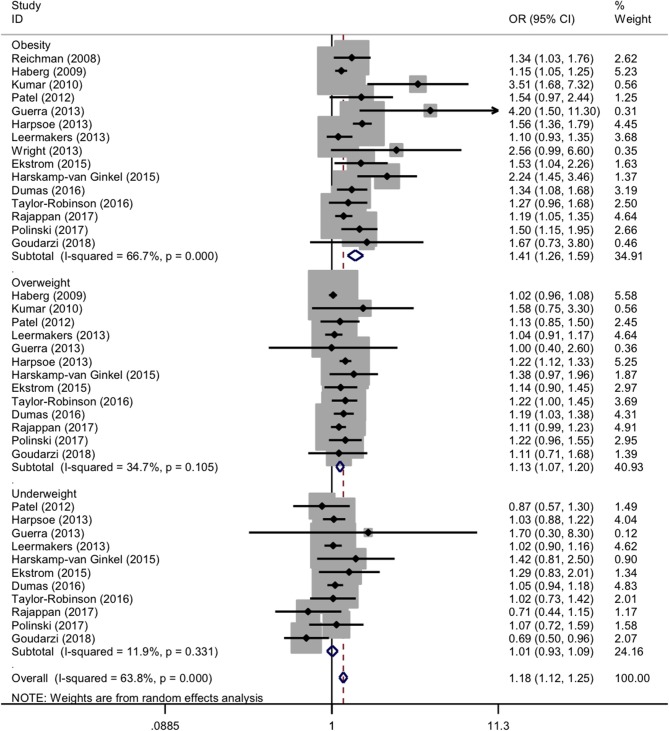
Forest plots for categorical body mass index after adjustment. OR, odds ratio; 95% CI, 95% confidence interval. The gray shadow size represents the proportion of the weight. The black line equal to 1 perpendicular to the horizontal axis represents an invalid line, and the red dashed line parallel to the black line represents the combined effect line of all the included studies.

For maternal categorical BMI in pre-pregnancy, the forest plots of study outcomes before and after adjustment are shown in Supplementary Figure 1 and Figure 2, respectively. Before adjustment, maternal obesity and overweight in pre-pregnancy significantly increased the risk of childhood asthma and wheeze (OR: 1.59 and 1.18, 95% CI: 1.38-1.83 and 1.06-1.3, both *p* < 0.001), with significant heterogeneity (*I*^2^: 79.5% and 78%; *p* < 0.001 and *p*: 0.002). The association between pre-pregnancy maternal underweight and the risk of childhood asthma and wheeze was statistically significant (OR: 1.09, 95% CI: 1.01-1.08, *p* < 0.001), without evidence of heterogeneity (*I*^2^: 0.0%; *p*: 0.486).

After adjustment, maternal obesity and overweight in pre-pregnancy significantly increased the risk of both childhood asthma and wheeze (OR: 1.41 and 1.13, 95% CI: 1.26-1.59 and 1.07-1.20, both *p* < 0.001), with moderate and low evidence of heterogeneity (*I*^2^: 66.7% and 34.7%; *p* < 0.001 and *p*: 0.105). Contrastingly, the association between maternal underweight in pre-pregnancy and the risk of childhood asthma and wheeze was nonsignificant (OR: 1.01, 95% CI: 0.93-1.09, *p*: 0.799), without heterogeneity (*I*^2^: 11.9%; *p*: 0.331).

**Figure 3:**
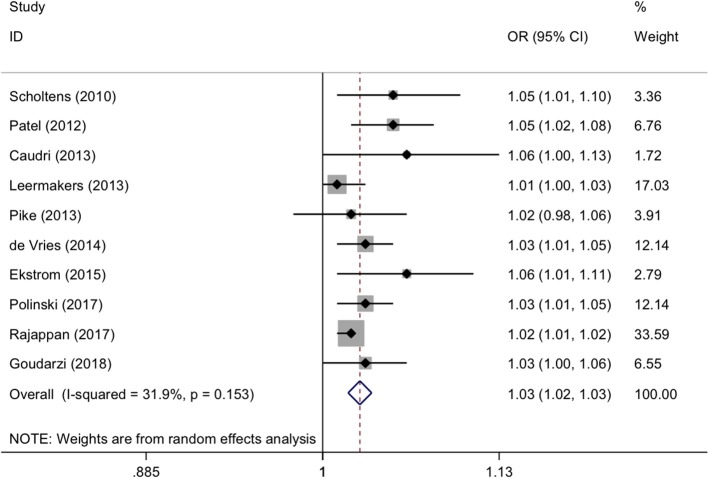
Forest plots for continuous body mass index after adjustment. OR, odds ratio; 95% CI, 95% confidence interval. The gray shadow size represents the proportion of the weight. The black line equal to 1 perpendicular to the horizontal axis represents an invalid line, and the red dashed line parallel to the black line represents the combined effect line of all the included studies.

For maternal continuous BMI in pre-pregnancy, the forest plots of study outcomes before and after adjustment are showed in Supplementary Figure 2 and Figure 3, respectively. Per 1 kg/m^2^ increment in maternal BMI was associated with a significantly increased risk of childhood asthma and wheeze before (OR: 1.03, 95% CI: 1.02-1.03, *p* < 0.001) and after (OR: 1.03, 95% CI: 1.02-1.03, *p* < 0.001) adjustment, with none and low evidence of heterogeneity (*I*^2^: 0.0% and 31.9%; *p*: 0.676 and 0.153, respectively).

**Figure 4:**
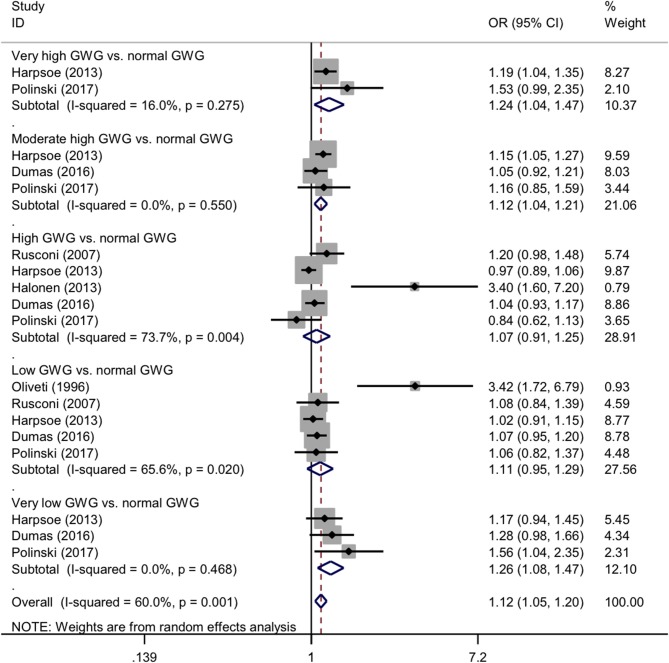
Forest plots for categorical gestational weight gain after adjustment. GWG, gestational weight gain; OR, odds ratio; 95% CI, 95% confidence interval. The gray shadow size represents the proportion of the weight. The black line equal to 1 perpendicular to the horizontal axis represents an invalid line, and the red dashed line parallel to the black line represents the combined effect line of all the included studies.

**Figure 5:**
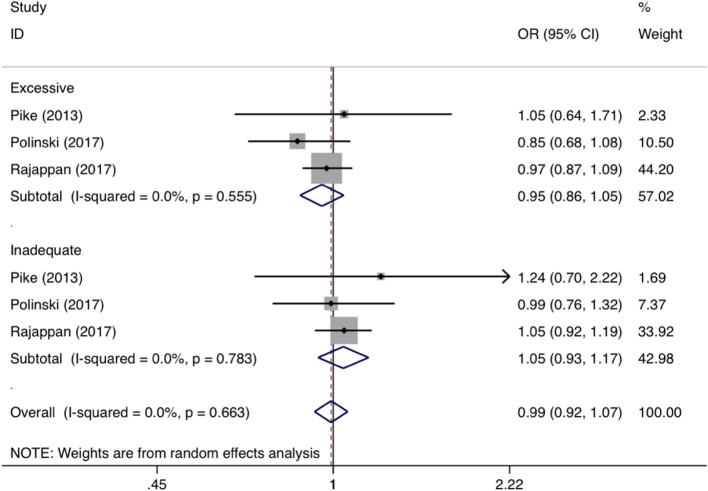
Forest plots for categorical gestational weight gain according to Institute of Medicine criteria after adjustment. OR, odds ratio; 95% CI, 95% confidence interval. The gray shadow size represents the proportion of the weight. The black line equal to 1 perpendicular to the horizontal axis represents an invalid line, and the red dashed line parallel to the black line represents the combined effect line of all the included studies.

For categorical GWG in pre-pregnancy, the forest plots of study outcomes before and after adjustment are shown in Supplementary Figure 3 and Figure 4, respectively. Before and after adjustment, the risk of childhood asthma and wheeze was significantly increased for very high GWG (OR: 1.38 and 1.24, 95% CI: 1.06-1.80 and 1.04-1.47, *p*: 0.016 and 0.018, *I*^2^: 47.4 and 18%), moderate high GWG (OR: 1.17 and 1.12, 95% CI: 1.08-1.26 and 1.04-1.21, *p* < 0.001 and *p*: 0.004, both *I*^2^: 0.0%), and very low GWG (OR: 1.67 and 1.26, 95% CI: 1.46-1.99 and 1.08-1.47, *p* < 0.001 and *p*: 0.004, *I*^2^: 27.1% and 0.0%), as compared with normal GWG. By contrast, high GWG was not associated with a significantly increased risk of childhood asthma and wheeze before (OR: 1.09, 95% CI: 0.90-1.31, *p*: 0.357, *I*^2^: 79.3%) and after (OR: 1.07, 95% CI:0.91-1.25, *p*: 0.493, *I*^2^: 73.7%) adjustment. Besides, there was significant association between low GWG (OR: 1.24, 95% CI: 1.03-1.50, *p*: 0.027, *I*^2^: 78.3%) and risk of asthma and wheeze in childhood before adjustment, yet no significant (OR: 1.11, 95% CI: 0.95-1.29, *p*: 0.182, *I*^2^: 65.6%) after adjustment. According to the Institute of Medicine criteria for GWG, the forest plots of study outcomes after adjustment are shown in Figure 5. Compared with adequate GWG, the risk of childhood asthma and wheeze was not significantly associated with inadequate GWG (OR: 0.95, 95% CI: 0.86-1.05, *p*: 0.44, *I*^2^: 0.0%), excessive GWG (OR: 0.99, 95% CI: 0.92-1.07, *p*: 0.31, *I*^2^: 0.0%).

### Subgroup Analyses

Due to significantly statistical heterogeneity encountered in overall analysis, several subgroup analyses were conducted separately according to sample size, region, weight modality, and diagnosis of asthma for maternal pre-pregnancy BMI (categorical and continuous) before (Supplementary Tables 4, 5) and after adjustment (Table 2 and Supplementary Table 6).

**Table 2:** Subgroup analysis for asthma/wheeze base on categorical BMI (adjusted).

**Group**	**Numbers** **(Asthma/Wheeze)**	**Asthma**		**Wheeze**	
		**OR (95% CI); *P***	***I*^**2**^**	**OR (95% CI); *P***	***I*^**2**^**
**Maternal obesity**					
By sample size					
Total sample size <6000	3/7	1.60 (1.20–2.13); 0.001	48.6%	1.68 (1.25–2.26); 0.001	73.0%
Total sample size ≥6000	3/6	1.50 (1.34–1.66); <0.001	0.0%	1.28 (1.10–149); 0.001	44.9%
By region					
America	3/2	1.39 (1.20–1.60); <0.001	0.0%	3.12 (1.74–5.13); <0.001	0.0%
Asia	NA/1	NA	NA	1.67 (0.73–3.81); 0.223	NA
Europe	3/10	1.63 (1.38–1.94); <0.001	19.4%	1.29 (1.14–1.45); <0.001	56.2%
By weight modality					
Medical records	2/4	1.40 (1.12–1.74); 0.003	0.0%	1.24 (1.00–1.55); 0.052	32.7%
Self-report	4/9	1.54 (1.34–1.77); <0.001	32.3%	1.48 (1.22–1.79); <0.001	70.4%
By diagnosis of asthma					
Doctor diagnosis	5/5	1.50 (1.33–1.69); <0.001	23.9%	1.48 (1.19–1.86); 0.001	36.1%
Parental report	1/8	1.53 (1.04–2.26); 0.032	NA	1.29 (1.11–1.51); 0.001	63.0%
**Maternal overweight**					
By sample size					
Total sample size <6000	4/8	1.19 (0.95–1.49); 0.123	47.1%	1.19 (0.95–1.49); 0.032	0.0%
Total sample size ≥6000	3/5	1.21 (1.13–1.30); <0.001	0.0%	1.21 (1.13–1.30); 0.022	41.9%
By region					
America	2/1	1.20 (1.06–1.38); 0.005	0.0%	1.58 (0.75–3.31); 0.226	NA
Asia	NA/1	NA	NA	1.11 (0.72–1.71); 0.635	NA
Europe	5/11	1.21 (1.06–1.37); 0.004	30.9%	1.07 (1.02–1.11); 0.003	0.0%
By weight modality					
Medical records	1/3	1.14 (0.90–1.45); 0.282	NA	1.12 (1.03–1.23); 0.013	0.0%
Self-report	6/10	1.21 (1.12–1.31); <0.001	10.6%	1.06 (1.01–1.12); 0.028	2.7%
By diagnosis of asthma					
Doctor diagnosis	4/4	1.22 (1.14–1.31); <0.001	0.0%	1.16 (1.03–1.30); 0.013	0.0%
Parental report	3/9	1.14 (0.86–1.51); 0.362	57.9%	1.06 (1.01–1.10); 0.019	0.0%

*BMI, body mass index; OR, odds ratio; 95% CI, 95% confidence interval; I^2^, inconsistency index; NA, not available*.

With regard to sample size, pre-pregnancy maternal obesity was associated with 50% increased risk of childhood asthma in studies with sample size ≥6000 (adjusted OR: 1.50, 95% CI: 1.34-1.66, *p* < 0.001), which was lower than studies with sample size < 6000 (adjusted OR: 1.60, 95% CI: 1.20-2.13, *p*: 0.001) (Table 2). The case was similar for the risk of childhood wheezing (adjusted OR: 1.28 and 1.68, 95% CI: 1.25-2.26 and 1.10-149, both *p*: 0.001 for studies with sample size ≥6000 and < 6000, respectively) (Table 2).

By diagnosis of asthma, pre-pregnancy maternal overweight was respectively associated with 22 and 14% increased risk of doctor-diagnosed asthma (adjusted OR: 1.22, 95% CI: 1.14-1.31, *p* < 0.001) and parent-reported asthma (adjusted OR: 1.14, 95% CI: 0.86-1.51, *p* < 0.362) (Table 2). By weight modality, the more significantly increased risk of asthma in childhood was detected in maternal obesity or overweight by self-report (adjusted OR: 1.54 and 1.21, 95% CI: 1.34-1.77 and 1.12-1.31, both *p* < 0.001) than by Medical records (adjusted OR: 1.40 and 1.14, 95% CI: 1.12-1.74 and 0.90-1.45, *p*: 0.003 and 0.282) (Table 2). By region, there was no obvious difference for the association between maternal pre-pregnancy obesity or overweight and the risk of childhood asthma (Table 2).

### Meta-Regression Analyses

To explore the extent to which study-level characteristics explained heterogeneity, meta-regression analyses were conducted by modeling averaged age and gender composition (Supplementary Figures 4, 5 for maternal categorical BMI in pre-pregnancy, Supplementary Figure 6 for maternal continuous BMI in pre-pregnancy). With the increase of male percentage, the risk of childhood wheeze associated with pre-pregnancy maternal obesity was significantly decreased before adjustment (*p*: 0.017), while no significance was reached after adjustment (*p*: 0.149). With the increase of averaged age, there was marginally statistical significance in the increased risk of childhood wheeze associated with pre-pregnancy maternal overweight before adjustment (*p*: 0.049), and there was no detectable significance after adjustment (*p*: 0.462).

### Publication Bias

The Begg's and filled funnel plots before and after adjustment are shown in Supplementary Figures 7, 8, respectively. Before adjustment, Begg's funnel plots seemed symmetrical for maternal continuous BMI, yet unsymmetrical for maternal categorical BMI and GWG, as confirmed by Egger's tests (*p*: 0.238, 0.003, and 0. 003, respectively). After adjustment, Begg's funnel plots seemed unsymmetrical in maternal BMI and GWG, as confirmed by Egger's tests (*p*: 0.011, 0.024, and 0. 003, respectively). As revealed by filled funnel plots, there were 3 to 12 estimated missing studies in the analysis of maternal BMI and GWG. Using the Duval and Tweedie “trim and fill” method to account for potential missing trials, significance was retained for unadjusted and adjusted maternal categorical BMI (OR: 1.14 and 1.121; 95% CI: 1.05-1.24 and 1.05-1.20; *p*: 0.002 and 0.001), as well as for unadjusted and adjusted maternal continuous BMI (OR: 1.02 and 1.02; 95% CI: 1.02-1.03 and 1.02-1.03; both *p* < 0.001), whereas no significance was seen for unadjusted and adjusted GWG (OR: 1.11 and 1.07; 95% CI: 1.00-1.23 and 0.99-1.16; *p*: 0.06 and 0.085). Additionally, we provided the scatter plots of sample size and publication year with effect size in Supplementary Figures 9, 10, respectively.

## Discussion

Via a comprehensive analysis of 145,574 mother-child pairs form 22 observational studies, our results support the hypothesis that both pre-pregnancy maternal obesity or overweight and very to moderate high or low GWG left their offspring susceptible to a significantly increased risk of having childhood asthma and wheeze. Moreover, as revealed by our subgroup analyses, sample size, weight modality, and diagnosis of asthma were potential sources of between-study heterogeneity. To the best of our knowledge, this is thus far the most comprehensive meta-analysis that has evaluated the association of pre-pregnancy maternal BMI and GWG with the risk of childhood asthma and wheeze.

Differing from the results of the previous meta-analysis by Forno and colleagues in 2014 (9), asthma and wheeze were analyzed both jointly and independently due to sufficient number of eligible studies in this present meta-analysis, and prespecified subgrouping of studies was not done according to ever and current asthma or wheeze due to ambiguous and inconsistent definitions across studies. Our results confirmed previously reported association of pre-pregnancy maternal obesity or overweight with an increased risk of having childhood asthma and wheeze in offspring found by Forno and colleagues (9). Besides, as an extension of previous studies, we observed that both very to moderate high and low GWG can significantly increase the risk of childhood asthma and wheeze. Our findings are biologically plausible. On one hand, obesity is established as a chronic and low-grade inflammatory state (35). There is evidence that obese women had higher serum proinflammatory markers than women with normal weight during pregnancy (36), and elevated inflammatory markers during pregnancy were closely linked to wheeze in offspring (37). Pregnancy per se is characterized by immunomodulatory changes (38), which along with obesity-driven proinflammatory state might affect fetal immune system development by placenta, and thus predispose the offspring to childhood asthma and wheeze (39, 40). On the other hand, diverse dietary patterns may contribute to the relation between maternal obesity and childhood asthma. For instance, maternal high-fat intake during pregnancy or meat intake before pregnancy was associated with an elevated risk of childhood asthma or wheeze (41, 42). In support of this association, experiment studies showed that asthma was a developmental origin disease influenced by maternal diets (43). Although a large number of studies have been conducted to explore and explain the association between maternal obesity and offspring asthma and wheeze, the exact mechanism of action underlying this association needs to be further elucidated. Nonetheless, from a public health viewpoint, our meta-analytical findings underscore the importance of mastering maternal weight gain in pre-pregnancy and during gestation and controlling them within a reasonable range to lower the future risk of suffering childhood asthma and wheeze in offspring.

It is worth noting that there was a dose-dependent relation between pre-pregnancy maternal weight and the risk for asthma and wheeze in childhood. In this meta-analysis, per pre-pregnancy BMI increment after adjustment was associated with an 3% increased risk of childhood asthma and wheeze, which was consistent with the result by Forno et al. (9). In support of this dose-dependent relation, adjusted pooled odds for childhood asthma and wheeze was increased from 1.13 for maternal overweight to 1.41 for maternal obesity in pre-pregnancy. Although the possibility of residual confounding cannot be fully eliminated, our findings seem reliable and robust, as effect-size estimates are still significant even after accounting for potentially missing studies as revealed by the trim-and-fill method.

Extending the findings of previous meta-analysis (9), we employed both subgroup and meta-regression analyses to seek the reasons for previously inconsistent results, and interestingly we found that sample size, weight modality, and diagnosis of asthma were potential sources of between-study heterogeneity. In particular, the association between pre-pregnancy maternal obesity or overweight and increased asthma and wheeze risk in this meta-analysis was more obvious in small studies than large studies, as well as in studies with doctor-diagnosed asthma than studies with parent-reported asthma, and such association was epidemiologically plausible. The significance in studies with large sample sizes reinforced the robustness of our principal finding. As for the diagnosis of asthma, a cross-sectional and longitudinal epidemiological survey revealed that the prevalence of asthma was 15.5% according to parental reports, and 21.5% according to doctor's diagnosis (44), indicating that asthma reported by parents may underestimate the risk of childhood asthma and wheeze related to asthma diagnosed by doctors. Contrastingly, there was no detectable significance in meta-regression analysis, likely due to its lack of the methodological rigor (45). It is expected that analysis of individual participants' data could make up methodological drawbacks and yield further insights, which is not practically feasible.

Despite the clear strengths of this meta-analysis, including large sample sizes, careful assessment of maternal weight, and comprehensive explorations on between-study heterogeneity, some possible limitations should be acknowledged when interpreting our findings. Firstly, only five pubic databases were reviewed for literature search, and this meta-analysis merely focused on articles published in the English language, which might yield a selection bias. Additionally, a majority of involved studies were longitudinal in design and with varied follow-up intervals, which may not capture the event of interest especially for studies with short follow-ups. Secondly, analysis on categorical GWG was based on only six studies, which precluded further subgroup analyses. Thirdly, in this meta-analysis, the categorization of asthma, such as ever, current, and persistent asthma was lack. Fourthly, some, but not all included studies had adjusted for child's weight or BMI at the time of assessment, indicating that the effect of maternal obesity on asthma and wheeze is not independent of childhood obesity, which is an established risk factor for asthma and wheeze (46).

Taken together, our findings indicate that both pre-pregnancy maternal obesity or overweight and very to moderate high or low GWG left their offspring susceptible to a significantly increased risk of having childhood asthma and wheeze. Moreover, sample size and diagnosis of asthma were potential sources of between-study heterogeneity. These data suggest that maintenance of maternal weight gain in pre-pregnancy and during gestation needs to be implemented as a primary prevention of the future development of childhood asthma and wheeze in offspring.

## Data Availability Statement

The datasets analyzed in this article are not publicly available. Requests to access the datasets should be directed to zhangzhixin032@163.com.

## Author Contributions

ZZ and WN conceived and designed the experiments. SL and BZ performed the experiments. SL and WN analyzed the data and wrote the paper. SL, YW, KW, and BZ contributed materials/analysis tools. All authors read and approved the final manuscript prior to submission.

### Conflict of Interest

The authors declare that the research was conducted in the absence of any commercial or financial relationships that could be construed as a potential conflict of interest.
