# Supplementary material for: Pre-pregnancy Maternal Weight and Gestational Weight Gain Increase the Risk for Childhood Asthma and Wheeze: An Updated Meta-Analysis
Source: Front Pediatr. 2020 Apr 3;8:134. doi: 10.3389/fped.2020.00134 (PMC7145976; doi:10.3389/fped.2020.00134)

**Supplementary Table 1.** The PRISMA checklist.

| **Section/topic** | **#** | **Checklist item** | **Reported on page #** |
| --- | --- | --- | --- |
| **TITLE** | | |  |
| Title | 1 | Identify the report as a systematic review, meta-analysis, or both. | 1 |
| **ABSTRACT** | | |  |
| Structured summary | 2 | Provide a structured summary including, as applicable: background; objectives; data sources; study eligibility criteria, participants, and interventions; study appraisal and synthesis methods; results; limitations; conclusions and implications of key findings; systematic review registration number. | 2-3 |
| **INTRODUCTION** | | |  |
| Rationale | 3 | Describe the rationale for the review in the context of what is already known. | 4-5 |
| Objectives | 4 | Provide an explicit statement of questions being addressed with reference to participants, interventions, comparisons, outcomes, and study design (PICOS). | 4-5 |
| **METHODS** | | |  |
| Protocol and registration | 5 | Indicate if a review protocol exists, if and where it can be accessed (e.g., Web address), and, if available, provide registration information including registration number. | 5 |
| Eligibility criteria | 6 | Specify study characteristics (e.g., PICOS, length of follow-up) and report characteristics (e.g., years considered, language, publication status) used as criteria for eligibility, giving rationale. | 5-6 |
| Information sources | 7 | Describe all information sources (e.g., databases with dates of coverage, contact with study authors to identify additional studies) in the search and date last searched. | 5-6 |
| Search | 8 | Present full electronic search strategy for at least one database, including any limits used, such that it could be repeated. | 5-6 |
| Study selection | 9 | State the process for selecting studies (i.e., screening, eligibility, included in systematic review, and, if applicable, included in the meta-analysis). | 5-6 |
| Data collection process | 10 | Describe method of data extraction from reports (e.g., piloted forms, independently, in duplicate) and any processes for obtaining and confirming data from investigators. | 6 |
| Data items | 11 | List and define all variables for which data were sought (e.g., PICOS, funding sources) and any assumptions and simplifications made. | 6-7 |
| Risk of bias in individual studies | 12 | Describe methods used for assessing risk of bias of individual studies (including specification of whether this was done at the study or outcome level), and how this information is to be used in any data synthesis. | 7-8 |
| Summary measures | 13 | State the principal summary measures (e.g., risk ratio, difference in means). | 7-8 |
| Synthesis of results | 14 | Describe the methods of handling data and combining results of studies, if done, including measures of consistency (e.g., I^2^) for each meta-analysis. | 7-8 |
| Risk of bias across studies | 15 | Specify any assessment of risk of bias that may affect the cumulative evidence (e.g., publication bias, selective reporting within studies). | 7-8 |
| Additional analyses | 16 | Describe methods of additional analyses (e.g., sensitivity or subgroup analyses, meta-regression), if done, indicating which were pre-specified. | 7-8 |
| **RESULTS** | | |  |
| Study selection | 17 | Give numbers of studies screened, assessed for eligibility, and included in the review, with reasons for exclusions at each stage, ideally with a flow diagram. | 9 |
| Study characteristics | 18 | For each study, present characteristics for which data were extracted (e.g., study size, PICOS, follow-up period) and provide the citations. | 9 |
| Risk of bias within studies | 19 | Present data on risk of bias of each study and, if available, any outcome level assessment (see item 12). | 9-10 |
| Results of individual studies | 20 | For all outcomes considered (benefits or harms), present, for each study: (a) simple summary data for each intervention group (b) effect estimates and confidence intervals, ideally with a forest plot. | 9-11 |
| Synthesis of results | 21 | Present results of each meta-analysis done, including confidence intervals and measures of consistency. | 9-11 |
| Risk of bias across studies | 22 | Present results of any assessment of risk of bias across studies (see Item 15). | 13 |
| Additional analysis | 23 | Give results of additional analyses, if done (e.g., sensitivity or subgroup analyses, meta-regression [see Item 16]). | 11-14 |
| **DISCUSSION** | | |  |
| Summary of evidence | 24 | Summarize the main findings including the strength of evidence for each main outcome; consider their relevance to key groups (e.g., healthcare providers, users, and policy makers). | 14-16 |
| Limitations | 25 | Discuss limitations at study and outcome level (e.g., risk of bias), and at review-level (e.g., incomplete retrieval of identified research, reporting bias). | 18 |
| Conclusions | 26 | Provide a general interpretation of the results in the context of other evidence, and implications for future research. | 18-19 |
| **FUNDING** | | |  |
| Funding | 27 | Describe sources of funding for the systematic review and other support (e.g., supply of data); role of funders for the systematic review. | Not available |

*From:* Moher D, Liberati A, Tetzlaff J, Altman DG, The PRISMA Group (2009). Preferred Reporting Items for Systematic Reviews and Meta-Analyses: The PRISMA Statement. PLoS Med 6(6): e1000097. doi:10.1371/journal.pmed1000097.

**Supplementary Table 2.** Quality Assessment of Included case-control studies in This Meta-analysis.

| **Author (year)** | **Q1** | **Q2** | **Q3** | **Q4** | **Q5** | **Q6** | **Q7** | **Q8** | **Star** |
| --- | --- | --- | --- | --- | --- | --- | --- | --- | --- |
| **Oliveti (1996)** | 1 | 1 | 0 | 1 | 2 | 0 | 1 | 0 | 6 |
| **Rusconi (2007)** | 0 | 0 | 1 | 1 | 1 | 0 | 1 | 0 | 4 |

The Newcastle-Ottawa scale for case-control studies (from Stang A. Eur J Epidemiol. 2010;25(9):603-605):

Note: A study can be awarded a maximum of one star for each numbered item within the Selection and Exposure categories. A maximum of two stars can be given for Comparability.

**Selection:**

**Q1.** Is the case definition adequate?

**Q2.** Representativeness of the cases

**Q3.** Selection of Controls

**Q4.** Definition of Controls

**Comparability:**

**Q5.** Comparability of cases and controls on the basis of the design or analysis

**Exposure:**

**Q6.** Ascertainment of exposure

**Q7.** Same method of ascertainment for cases and controls

**Q8.** Non-Response rate

**Supplementary Table 3.** Quality Assessment of Included cohort studies in This Meta-analysis.

| **Author (year)** | **Q1** | **Q2** | **Q3** | **Q4** | **Q5** | **Q6** | **Q7** | **Q8** | **Star** |
| --- | --- | --- | --- | --- | --- | --- | --- | --- | --- |
| **Reichman (2008)** | 1 | 1 | 1 | 1 | 1 | 1 | 1 | 0 | 7 |
| **Haberg (2009)** | 1 | 1 | 0 | 1 | 1 | 0 | 1 | 0 | 5 |
| **Kumar (2010)** | 1 | 1 | 0 | 1 | 1 | 0 | 1 | 1 | 6 |
| **Scholtens (2010)** | 1 | 1 | 0 | 1 | 1 | 0 | 1 | 1 | 6 |
| **Patel (2012)** | 1 | 1 | 1 | 1 | 1 | 1 | 1 | 1 | 8 |
| **Caudri (2013)** | 1 | 1 | 0 | 1 | 1 | 0 | 1 | 1 | 6 |
| **Guerra (2013)** | 1 | 1 | 0 | 1 | 1 | 0 | 1 | 1 | 6 |
| **Halonen (2013)** | 1 | 1 | 1 | 1 | 1 | 1 | 1 | 1 | 8 |
| **Harpsoe (2013)** | 1 | 1 | 0 | 1 | 1 | 1 | 1 | 1 | 8 |
| **Leermakers (2013)** | 1 | 1 | 0 | 1 | 1 | 0 | 1 | 1 | 6 |
| **Pike (2013)** | 1 | 1 | 1 | 1 | 1 | 0 | 1 | 1 | 7 |
| **Wright (2013)** | 1 | 1 | 1 | 1 | 1 | 0 | 1 | 1 | 7 |
| **de Vries (2014)** | 1 | 1 | 0 | 1 | 1 | 0 | 1 | 1 | 6 |
| **Ekstrom (2015)** | 1 | 1 | 1 | 1 | 1 | 0 | 1 | 0 | 6 |
| **Harskamp-van (2015)** | 1 | 1 | 0 | 1 | 1 | 1 | 1 | 1 | 7 |
| **Dumas (2016)** | 1 | 1 | 0 | 1 | 1 | 1 | 1 | 0 | 6 |
| **Taylor-Robinson (2016)** | 1 | 1 | 0 | 1 | 1 | 0 | 1 | 1 | 6 |
| **Polinski (2017)** | 1 | 1 | 0 | 1 | 1 | 1 | 1 | 0 | 6 |
| **Rajappan (2017)** | 1 | 1 | 1 | 1 | 1 | 0 | 1 | 0 | 6 |
| **Goudarzi (2018)** | 1 | 1 | 0 | 1 | 1 | 0 | 1 | 0 | 6 |

The Newcastle-Ottawa scale for cohort studies (from Stang A. Eur J Epidemiol. 2010;25(9):603-605):

Note: A study can be awarded a maximum of one star for each numbered item within the Selection and Outcome categories. A maximum of two stars can be given for Comparability

**Selection:**

**Q1.** Representativeness of the exposed cohort

**Q2.** Selection of the non exposed cohort

**Q3.** Ascertainment of exposure

**Q4.** Demonstration that outcome of interest was not present at start of study

**Comparability:**

**Q5.** Comparability of cohorts on the basis of the design or analysis

**Outcome:**

**Q6.** Assessment of outcome

**Q7.** Was follow-up long enough for outcomes to occur

**Q8.** Adequacy of follow up of cohorts

**Supplementary Table 4.** Subgroup analysis for asthma/wheeze based on categorical BMI (unadjusted).

| **Group** | **Numbers**  **(Asthma/Wheeze)** | **Asthma** | | **Wheeze** | |
| --- | --- | --- | --- | --- | --- |
|  |  | **OR (95% CI); P** | ***I*^2^** | **OR (95% CI); P** | ***I*^2^** |
| **Maternal obesity**  By sample size |  |  |  |  |  |
| Total sample size <6000 | 2/5 | 1.99 (1.12-3.53); 0.019 | 83.9% | 2.24 (1.31-3.83); 0.003 | 81.7% |
| Total sample size ≥6000 | 3/6 | 1.60 (1.45-1.77); <0.001 | 0.0% | 1.38 (1.17-1.61); <0.001 | 59.4% |
| By region |  |  |  |  |  |
| America | 3/2 | 1.58 (1.38-1.81); <0.001 | 0.0% | 2.99 (1.74-5.13); <0.001 | 0.0% |
| Europe | 2/9 | 2.02 (1.2-3.38); 0.008 | 84.4% | 1.41 (1.23-1.62); <0.001 | 67.3% |
| By weight modality |  |  |  |  |  |
| Medical records or measure | 1/4 | 1.51 (1.18-1.93); 0.001 | NA | 1.26 (1.03-1.54); 0.022 | 39.5% |
| Self-report | 4/7 | 1.74 (1.45-2.09); <0.001 | 62.1% | 1.74 (1.35-2.24); <0.001 | 78.7% |
| By diagnosis of asthma |  |  |  |  |  |
| Doctor diagnosis | NA /5 | NA | NA | 1.55 (1.24-1.94); <0.001 | 49.7% |
| Parental report | NA /6 | NA | NA | 1.45 (1.18-1.73); <0.001 | 73.4% |
| **Maternal overweight** |  |  |  |  |  |
| By sample size |  |  |  |  |  |
| Total sample size <6000 | 3/6 | 1.31 (0.98-1.77); 0.073 | 56.4% | 1.10 (1.00-1.20); 0.044 | 0.0% |
| Total sample size ≥6000 | 3/5 | 1.25 (1.17-1.33); <0.001 | 0.0% | 1.19 (1.00-1.40); 0.046 | 85.3% |
| By region |  |  |  |  |  |
| America | 2/1 | 1.27 (1.12-1.43); <0.001 | 0.0% | 1.48 (0.72-3.06); 0.288 | NA |
| Europe | 4/10 | 1.27 (1.09-1.49); 0.002 | 38.3% | 1.16 (1.04-1.29); 0.008 | 71.4% |
| By weight modality |  |  |  |  |  |
| Medical records or measure | NA /3 | NA | NA | 1.18 (1.03-1.34); 0.014 | 49.3% |
| Self-report | NA /8 | NA | NA | 1.16 (0.99-1.36); 0.065 | 69.5% |
| By diagnosis of asthma |  |  |  |  |  |
| Doctor diagnosis | 4/4 | 1.25 (1.17-1.31); <0.001 | 0.0% | 1.25 (1.13-1.39); <0.001 | 0.0% |
| Parental report | 2/7 | 1.25 (1.18-1.33); 0.318 | 78.1% | 1.12 (0.98-1.27); 0.088 | 62.9% |

Abbreviations: BMI, body mass index; OR, odds ratio; 95% CI, 95% confidence interval; I^2^, inconsistency index; NA, not available.

**Supplementary Table 5.** Subgroup analysis for asthma/wheeze based on continuous BMI (unadjusted).

| **Group** | **Numbers**  **(Asthma/Wheeze)** | **Asthma** | | **Wheeze** | |
| --- | --- | --- | --- | --- | --- |
|  |  | **OR (95% CI); P** | ***I*^2^** | **OR (95% CI); P** | ***I*^2^** |
| By total sample size |  |  |  |  |  |
| Total sample size <3000 | 2/4 | 1.01 (0.99-1.04); 0.263 | 0.0% | 1.02 (1.01-1.03); <0.001 | 0.0% |
| Total sample size ≥3000 | 1/4 | 1.03 (1.02-1.05); <0.001 | NA | 1.03 (1.02-1.04); <0.001 | 0.0% |
| By region |  |  |  |  |  |
| America | 1/ NA | 1.03 (1.02-1.05); <0.001 | NA | NA | NA |
| Asia | NA /1 | NA | NA | 1.03 (1.00-1.06); 0.047 | NA |
| Europe | 2/7 | 1.01 (0.99-1.04); 0.263 | 0.0% | 1.02 (1.02-1.03); <0.001 | 0.0% |
| By weight modality |  |  |  |  |  |
| Medical records or measure | 2/6 | 1.01 (0.99-1.04); 0.159 | 0.0% | 1.02 (1.02-1.03); <0.001 | 6.5% |
| Self-report | 1/2 | 1.03 (1.02-1.05); 0.238 | NA | 1.03 (1.01-1.05); <0.001 | 0.0% |
| By diagnosis of asthma |  |  |  |  |  |
| Doctor diagnosis | 1/2 | 1.03 (1.02-1.05); <0.001 | NA | 1.03 (1.02-1.05); <0.001 | 0.0% |
| Parental report | NA /3 | NA | NA | 1.02 (1.01-1.03); <0.001 | 0.0% |
| Both | 2/3 | 1.01 (0.99-1.04); 0.263 | 0.0% | 1.02 (1.01-1.03); 0.005 | 0.0% |

Abbreviations: BMI, body mass index; OR, odds ratio; 95% CI, 95% confidence interval; I^2^, inconsistency index; NA, not available.

**Supplementary Table 6.** Subgroup analysis for asthma/wheeze based on continuous BMI (adjusted)

| **Group** | **Numbers**  **(Asthma/Wheeze)** | **Asthma** | | **Wheeze** | |
| --- | --- | --- | --- | --- | --- |
|  |  | **OR (95% CI); P** | ***I*^2^** | **OR (95% CI); P** | ***I*^2^** |
| By sample size |  |  |  |  |  |
| Total sample size <3000 | 2/4 | 1.01 (0.98-1.04); 0.511 | 0.0% | 1.02 (1.01-1.02); <0.001 | 26.3% |
| Total sample size ≥3000 | 4/9 | 1.03 (1.00-1.06); 0.059 | 61.9% | 1.02 (1.01-1.03); <0.001 | 39.0% |
| By region |  |  |  |  |  |
| America | 1/NA | 1.03 (1.01-1.05); 0.003 | NA | NA | NA |
| Asia | NA/1 | NA | NA | 1.03 (1.00-1.06); 0.047 | NA |
| Europe | 5/12 | 1.02 (0.99-1.05); 0.164 | 56.5% | 1.02 (1.01-1.03); <0.001 | 37.1% |
| By weight modality |  |  |  |  |  |
| Medical records | 3/6 | 1.02 (0.99-1.06); 0.159 | 47.2% | 1.02 (1.01-1.03); <0.001 | 48.8% |
| Self-report | 3/7 | 1.02 (0.99-1.06); 0.238 | 67.3% | 1.02 (1.01-1.03); <0.001 | 27.0% |
| By diagnosis of asthma |  |  |  |  |  |
| Doctor diagnosis | 1/2 | 1.03 (1.01-1.05); 0.003 | NA | 1.01 (1.00-1.02); <0.001 | 16.0% |
| Parental report | 3/8 | 1.03 (0.98-1.08); 0.268 | 74.4% | 1.04 (1.02-1.06); <0.001 | 16.5% |
| Both | 2/3 | 1.01 (0.98-1.04); 0.511 | 0.0% | 1.02 (1.01-1.03); 0.020 | 0.0% |

Abbreviations: BMI, body mass index; OR, odds ratio; 95% CI, 95% confidence interval; I^2^, inconsistency index; NA, not available.

**Supplementary Table 7.** P values and Egger’s tests before and after adjustment.

| **Groups** | **P value**  **(unadjusted)** | **P value**  **(adjusted)** | **Egger's test**  **(unadjusted)** | **Egger's test**  **(adjusted)** |
| --- | --- | --- | --- | --- |
| Categorical BMI |  |  | 0.003 | 0.024 |
| Obesity | <0.001 | <0.001 |  |  |
| Overweight | <0.001 | <0.001 |  |  |
| Underweight | <0.001 | 0.799 |  |  |
| Continuous BMI | <0.001 | 0.001 | 0.238 | 0.011 |
| Categorical GWG |  |  | 0. 003 | 0.003 |
| Very high GWG | 0.016 | 0.018 |  |  |
| Moderate high GWG | 0.001 | 0.004 |  |  |
| High GWG | 0.357 | 0.493 |  |  |
| Low GWG | 0.027 | 0.182 |  |  |
| Very low GWG | 0.001 | 0.004 |  |  |
| Categorical GWG (IOM) |  |  |  |  |
| Inadequate GWG | NA | 0.44 |  |  |
| Excessive GWG | NA | 0.31 |  |  |

Abbreviations: BMI, body mass index; GWG, gestational weight gain; IOM, Institute of Medicine; NA, not available.

**Supplementary Figure 1.** Forest plots for categorical body mass index before adjustment. Abbreviations: OR, odds ratio; 95% CI, 95% confidence interval. The grey shadow size represents the proportion of the weight. The black line equal to 1 perpendicular to the horizontal axis represents an invalid line, and the red dashed line parallel to the black line represents the combined effect line of all the included studies.

**Supplementary Figure 2.** Forest plots for continuous body mass index before adjustment. Abbreviations: OR, odds ratio; 95% CI, 95% confidence interval. The grey shadow size represents the proportion of the weight. The black line equal to 1 perpendicular to the horizontal axis represents an invalid line, and the red dashed line parallel to the black line represents the combined effect line of all the included studies.

**Supplementary Figure 3.** Forest plots for categorical gestational weight gain before adjustment. Abbreviations: GWG gestational weight gain; OR, odds ratio; 95% CI, 95% confidence interval. The grey shadow size represents the proportion of the weight. The black line equal to 1 perpendicular to the horizontal axis represents an invalid line, and the red dashed line parallel to the black line represents the combined effect line of all the included studies.

**Supplementary Figure 4.** Meta-regression analyses for maternal pre-pregnancy obesity for asthma on averaged age (pane A and B) and male ratio (pane C and D), maternal pre-pregnancy obesity for wheeze on averaged age (pane E and F) and male ratio (pane G and H) before and after adjustment. Abbreviations: OR, odds ratio; 95% CI, 95% confidence interval. Blue solid circles represent effect-size estimates of individual studies, and pink vertical lines represent 95% CI of these effect-size estimates. The green dotted line represents the fitted regression line.

A B

C D

E F


G H

**Supplementary Figure 5.** Meta-regression analyses for maternal pre-pregnancy overweight for asthma on averaged age (pane A and B) and male ratio (pane C and D), maternal pre-pregnancy overweight for wheeze on averaged age (pane E and F) and male ratio (pane G and H) before and after adjustment. Abbreviations: OR, odds ratio; 95% CI, 95% confidence interval. Blue solid circles represent effect-size estimates of individual studies, and pink vertical lines represent 95% CI of these effect-size estimates. The green dotted line represents the fitted regression line.

A B

C D

E F


G H

**Supplementary Figure 6.** Meta-regression analyses for continuous body mass index for asthma on averaged age (pane A and B) and male ratio (pane C and D), continuous body mass index for wheeze on averaged age (pane E and F) and male ratio (pane G and H) before and after adjustment. Abbreviations: OR, odds ratio; 95% CI, 95% confidence interval. Blue solid circles represent effect-size estimates of individual studies, and pink vertical lines represent 95% CI of these effect-size estimates. The green dotted line represents the fitted regression line.

A B

C D

E F

G H

**Supplementary Figure 7.** Begg’s and filled funnel plots for categorical body mass index (pane A and B), continuous body mass index (pane C and D), and categorical gestational weight gain (pane E and F) before adjustment. Hollow circles represent all eligible studies in this meta-analysis, and solid squares represent potentially missing studies required to achieve symmetry.

A B

C D

E F

**Supplementary Figure 8.** Begg’s and filled funnel plots for categorical body mass index (pane A and B), continuous body mass index (pane C and D), and categorical gestational weight gain (pane E and F) after adjustment. Hollow circles represent all eligible studies in this meta-analysis, and solid squares represent potentially missing studies required to achieve symmetry.

A B

C D

E F

**Supplementary Figure 9.** The scatter plot of sample size for maternal categorical BMI (panel A and B), maternal continuous BMI (panel C and D), and categorical GWG (panel E and F) before and after adjustment.


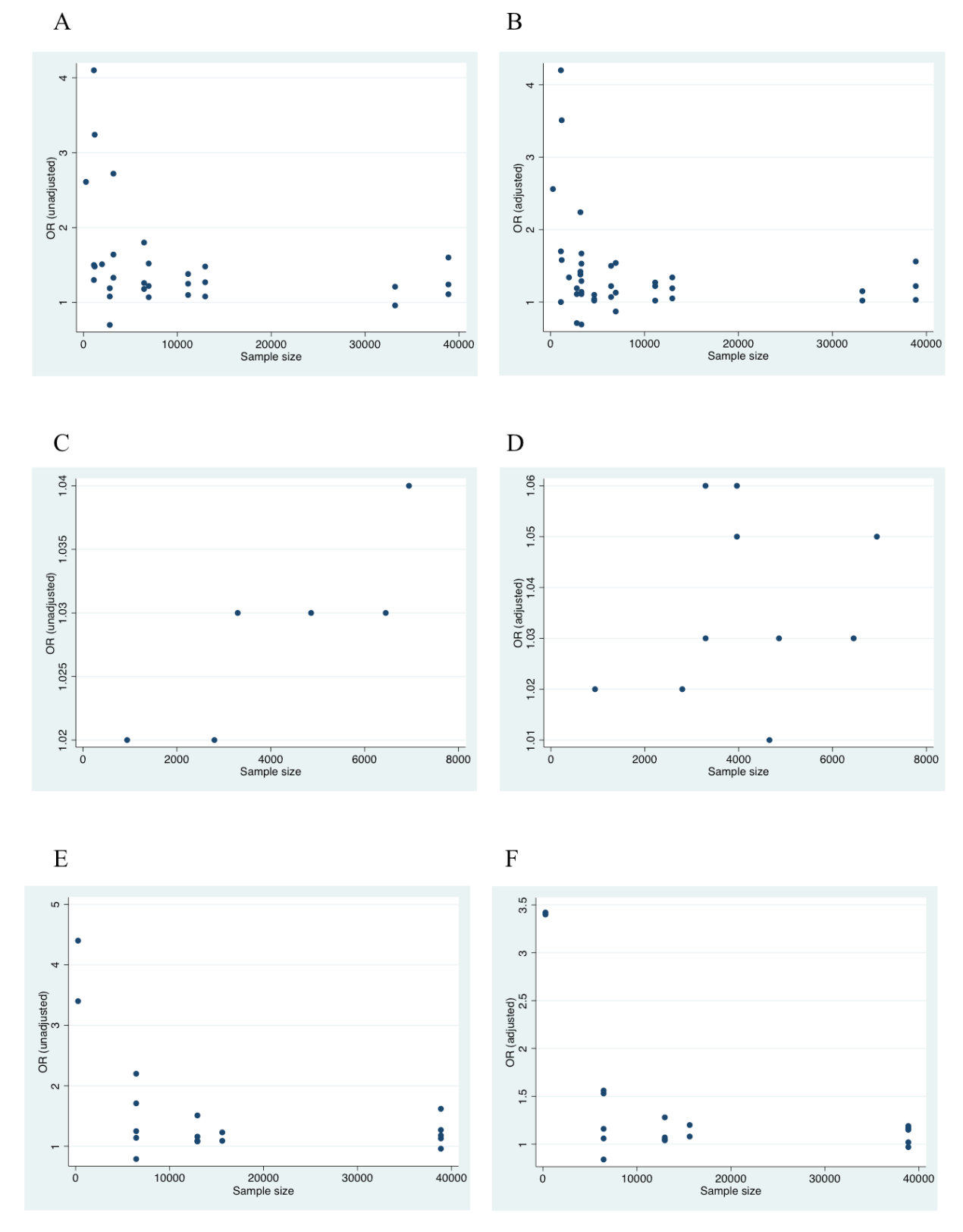


**Supplementary Figure 10.** The scatter plot of publication year for maternal categorical BMI (panel A and B), maternal continuous BMI (panel C and D), and categorical GWG (panel E and F) before and after adjustment.


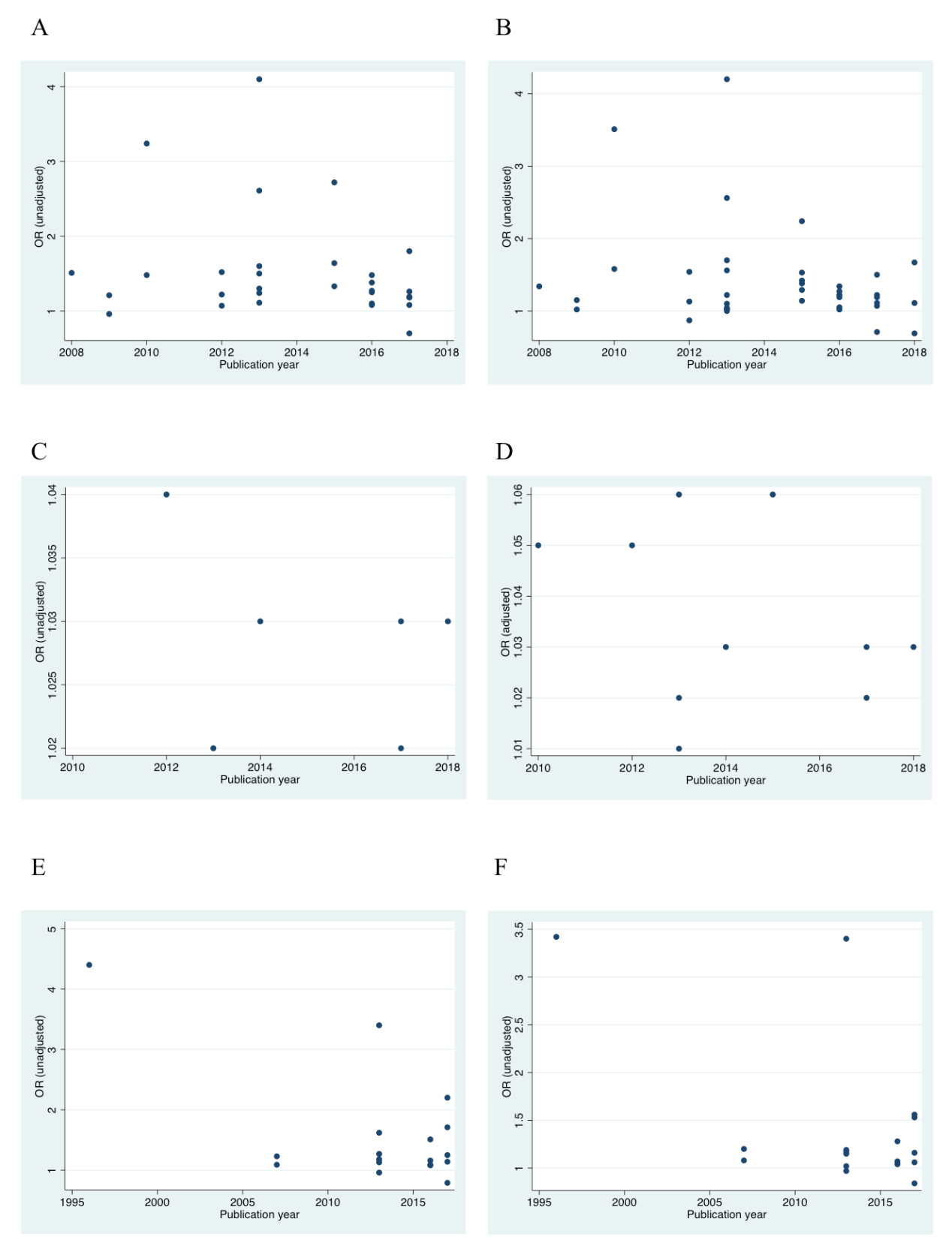

Supplement: Supplementary file 1 [file Data_Sheet_1.docx]
